# Interferon α2 and interferon γ induce the degranulation independent production of VEGF‐A and IL‐1 receptor antagonist and other mediators from human mast cells

**DOI:** 10.1002/iid3.211

**Published:** 2017-12-13

**Authors:** Sharon A. Oldford, Suzanne P. Salsman, Liliana Portales‐Cervantes, Raidan Alyazidi, Robert Anderson, Ian D Haidl, Jean S. Marshall

**Affiliations:** ^1^ Dalhousie Inflammation Group Dalhousie University Halifax Nova Scotia Canada; ^2^ Department of Microbiology and Immunology Dalhousie University Halifax Nova Scotia Canada; ^3^ Canadian Center for Vaccinology IWK Health Centre Halifax Nova Scotia Canada; ^4^ Faculty of Medicine Department of Pediatrics King Abdulaziz University Jeddah Kingdom of Saudi Arabia; ^5^ Department of Pediatrics Dalhousie University Halifax Nova Scotia Canada; ^6^ Department of Pathology Dalhousie University Halifax Nova Scotia Canada

**Keywords:** CXCL10, IL‐1Ra, interferons, mast cells, reovirus, respiratory syncytial virus, VEGF

## Abstract

**Background:**

Mast cells are resident immune effector cells, often studied in the context of allergic disease. Found in substantial numbers at sites of potential infection they are increased at sites of angiogenesis and can be pivotal for the sensing and clearance of a variety of pathogens. Interferons (IFNs) are cytokines that are critical for host defence against intracellular pathogens. Increased levels of IFNs are observed during viral infection and in autoimmune diseases. IFNs are also widely used therapeutically and have been examined in the therapy of severe asthma.

**Objective:**

To define the selective human mast cell cytokine and chemokine response following activation with type I or type II IFN's.

**Methods:**

The ability of both IFNα2 and IFNγ to induce cytokine production by human cord blood‐derived mast cells was examined in vitro. Cytokine and chemokine production at 6 and 24 h was assessed by multiplex protein analysis. Degranulation was assessed by β‐hexosaminidase release. Mast cells were also treated with reovirus or respiratory syncytial virus and their production of CXCL10, IL‐1 receptor antagonist (IL‐1Ra), and vascular endothelial growth factor (VEGF) examined after 24 h.

**Results:**

In addition to increased expression of classical IFN response genes, such as CXCL10, small but significant increases in CCL5 and IL‐17 production were observed following IFN activation. Notably, human mast cells produced both VEGF and IL‐1Ra in a dose dependent manner. These responses occurred in the absence of mast cell degranulation by a mechanism consistent with classical IFN signaling. Both reovirus and respiratory syncytial virus infection of mast cells, were also associated with IFN‐dependent IL‐1Ra expression.

**Conclusion and Clinical Relevance:**

Our findings demonstrate that IFNs have profound impact on cytokine and chemokine expression by human mast cells, alone or in the context of viral infection. Mast cell VEGF and IL‐1Ra responses to IFNs could impact the regulation of local inflammatory responses and subsequent tissue remodeling.

## Introduction

Mast cells are abundant at mucosal sites and skin sites. They have been extensively studied for their role in allergic disease. However, they also play important roles in tissue remodeling and host defense 1. Mast cells are key regulators of tissue homeostasis through interactions with stromal cells, such as endothelial cells, fibroblasts, and myofibroblasts and their release of tissue degrading proteases 2. Activated mast cells enhance endothelial cell migration, proliferation, and blood vessel formation through release of multiple mediators including VEGF‐A, FGF‐2, TGF‐β, TNF, and CXCL8 and can further promote angiogenesis via release of proteases that release extracellular matrix bound pro‐angiogenic factors 3. Traditionally, mast cell activation is associated with the release of pre‐formed granule mediators. However, activation and mediator production in response to many pathogen associated and cytokine stimuli can often occur without concurrent degranulation 1.

Mast cells are critical sentinel cells that mobilize immune effector cells to sites of infection and interact with innate and adaptive immune cells to modulate immunity 1, 4. Mast cells release a wide variety of de novo synthesized cytokines and chemokines in response to a number of non‐degranulating stimuli such as TLR activators 5, 6, 7. Resident in large numbers at sites that interface the environment, such as the skin, gastrointestinal, and respiratory mucosae 1, 4, 8, mast cells are poised to act as “first responders” following pathogen entry and help facilitate the mobilization of effective immunity though the generation of cytokines and chemokines. In viral infection such responses can also be detrimental by enhancing vascular leakage 9. Mast cells additionally play an important role in inflammation resolution at tissue sites. Indeed, IgE‐activated human mast cells, can secrete IL‐1 receptor antagonist (IL‐1Ra) 10 and IL‐10 11 which inhibits the function of pro‐inflammatory IL‐1β. Such mast cell IL‐10 responses are further enhanced in the presence of type I interferon (IFN) 11.

IFNs are immune‐regulatory cytokines critical for host defense and the induction of effective innate and adaptive immune responses. Type I IFNs are released from stromal and immune cells, including mast cells, in response to pathogen exposure and mediate their effects via binding to the heterodimeric IFNA receptor (IFNAR) 12. Type II IFN (IFNγ) is mainly produced by activated NK cells and T cells and mediates its effects via binding to the heterodimeric IFN γ receptor (IFNGR) 12. All classes of IFN are important for the initiation and regulation of innate and adaptive immune responses 12, 13.

The importance of type I and type II IFNs in immunity are well illustrated by their clinical efficacy. IFNα2 has been used in the treatment of several cancers including bladder cancer 14 and melanoma 15. Type I interferons are routinely used in the treatment of multiple sclerosis 16 and IFNγ has gained regulatory approval for use in the treatment of chronic granulomatous disease and severe malignant osteopetrosis and has been used to treat invasive fungal infections, idiopathic pulmonary fibrosis, tuberculosis, and scleroderma 17. Type I IFNs are known to be central to anti‐viral host responses 18 and IFNα2 has been used for the treatment of chronic viral infections including hepatitis C virus 19, hepatitis B virus 20, and human papilloma virus 21. A clinical trial has also demonstrated efficacy for IFNβ in the treatment of virally‐induced asthma exacerbations in hard‐to‐treat asthmatics 22. To better understand immune modulation following recombinant IFN administration and the role of IFNs in local anti‐viral immunity at mucosal sites it is crucial to determine their effects on resident, sentinel, innate immune cells. Few studies have examined the interaction between mast cells and IFNs. However, an early report demonstrated that IFNγ can inhibit murine mast cell degranulation and lipid mediator production 23. IFNγ has also been shown to enhance Fcγ receptor expression 24, 25 and both inflammatory and bacteriocidal activities of human mast cells in response to Gram positive bacteria 26. In this study, we examined the impact of direct type I and type II IFN activation of human mast cells on cytokine and chemokine production relevant to inflammatory disease and the potential for selected IFN responses to be of importance in previously described human mast cell responses to viral infection 27, 28.

## Materials and Methods

### Human cord blood derived mast cells

Human umbilical cord blood was obtained following written consent and the data analyzed anonymously in accordance with the procedures recommended by the Izaak Walton Killam Health Center Research Ethics Board Halifax, NS, Canada (Approved protocol number: IWK HE 1005110). Human cord blood–derived mast cells (CBMC) were generated according to an adaptation of the methods described by Enoksson et al. 29. Briefly, mononuclear cells obtained from umbilical cord blood were cultured at 1.0 × 10^6^ cells/ml and passaged once per week for 4 weeks in StemSpan SFEM medium (Stem Cell Technologies, Vancouver, BC, Canada) containing 10 ng/ml hIL‐3 (present in the medium only the first week of culture, eBiosciences, San Diego, CA), 10 ng/ml hIL‐6 and 100 ng/ml hSCF (Peprotech, Rocky Hill, NJ) in 5% CO_2_ at 37°C in humidified atmosphere. At 5 weeks of culture, the medium was replaced by RPMI 1640 (Sigma, St Louis, MO) supplemented with 10% Fetal Bovine Serum (FBS; Sigma), 2 mM L‐glutamine (HyClone Labs, Rockford, IL), 100 U/ml penicillin G, 100 μg/ml streptomycin (HyClone), 5 × 10^−5 ^M 2‐mercaptoethanol (2‐ME, Sigma), 15 mM HEPES (HyClone), 0.1 mM non‐essential amino acids (HyClone), 100 ng/ml hSCF and 10 ng/ml hIL‐6. The purity of mast cells used in the experiments was >95% CD117^+^ as assessed by flow cytometry using anti‐CD117 (c‐kit)‐APC (clone 104D2, BioLegend, San Diego, CA) beginning at week 6. Mast cells produced in this way were on average 96.5% positive for CD117 (c‐kit), 99.3% positive for mast cell tryptase and less than 0.2% positive for a variety of other lineage markers including CD3, CD11b, CD19, CD56, CD86, CD163, and CD209.

### IFN activation of CBMC

For all IFN activations and virus infection experiments, CBMC were “rested” in low hSCF conditions (10 ng/ml) overnight in RPMI 1640 supplemented with 10% FBS, 2 mM L‐glutamine (HyClone), 100 U/ml penicillin G, 100 μg/ml streptomycin, 15 mM HEPES, 0.1 mM non‐essential amino acids, 10 ng/ml hSCF, and 10 ng/ml hIL‐6. For IFN activations, CBMC (10^6^/ml) were treated for 6, 12, or 24 h with of 0.05, 0.5, or 5 ng/ml (1, 10, or 100 IU/ml) hIFNγ (Peprotech), or with 0.01, 1, or 10 ng/ml (1, 10, or 100 manufacturer U/ml) hIFNα2a (US Biological, Salem, MA). Cells were activated in RPMI 1640 supplemented with 10 ng/ml hSCF, 1% FBS, 15 mM HEPES, 100 μg/ml soybean trypsin inhibitor and 20 μg/ml leupeptin (Sigma). In some experiments CBMC were pretreated for 1 h with 2 μM of the PI3 K inhibitor LY 294002, the p38‐MAPK inhibitor SB 203580 or with the JAK/STAT inhibitor JAK inhibitor 1 (EMD Millipore, Billerica, MA).

### Beta‐hexosaminidase assay

CBMC(1 × 10^6^/ml) in modified HEPES‐Tyrode's buffer were treated for 20 min with increasing doses of IFNγ or IFNα2 or calcium ionophore A23187 (0.5 μM), as a positive control. The level of degranulation was assessed via β‐hexosaminidase release according to the method of Schwartz et al.^30^

### RSV and reovirus activation of CBMC

Mammalian reovirus (type 3 Dearing) and RSV (serotype A, Long strain) were propagated, purified, and UV‐inactivated as previously described 28, 31, 32. UV inactivation was confirmed by standard plaque assay and flow cytometric analysis of virus treated cells. Reovirus adsorption was performed by incubating CBMCs at 5 × 10^6^ cells/ml with reovirus at 20 multiplicities of infection (MOI) in activation medium (RPMI 1640 containing 1% FBS, 15 mM HEPES and 10 ng/ml hSCF) for 1 h at 37°C. Medium alone (Mock) or UV‐Reo at MOI 20 were included as controls. The cells were washed and resuspended at 1 × 10^6^ cells/ml in fresh activation medium plus 1% FBS and 100 μg/ml soybean trypsin inhibitor (Sigma). RSV adsorption was performed by incubating CBMC (10^6^/ml) for 90 min at 4°C with RSV at MOI 3–4 in activation media containing 2.5% FBS and 100 μg/ml soybean trypsin inhibitor (Sigma). Medium alone (Mock) or UV‐RSV at MOI 3–4 were included as controls. In some experiments, CBMC were resuspended in activation media containing 5 μg/ml anti‐human anti‐IFN‐α/β receptor chain 2 antibody (clone MMHAR‐2, EMD Millipore) or mouse IgG2a isotype control (clone, MG2a‐53, BioLegend). Cell‐free supernatants were harvested by centrifugation. Cell pellets were lysed and stored for mRNA extraction.

### Reverse transcription quantitative PCR (RT‐qPCR)

RNA was extracted from CBMC following 24 h activation using the RNeasy Plus Mini Kit (Qiagen, Toronto, ON). RNA was depleted of any contaminating genomic DNA and converted to cDNA using the Quantitech Reverse Transcription kit (Qiagen), according to manufacturer's protocol. Commercial primer sets were used to quantify expression of *ISG56* (BioRad, Missisauga, ON), *MX1*, *IRF1*, and *CXCL10* (all from SABiosciences, Mississauga, ON). *GAPDH* (Invitrogen, Burlington, ON) and *HPRT1* (BioRad) were used as reference genes. The amplification efficiency of each primer pair was checked with serial dilutions of cDNA and calculated efficiency values (all with E‐values >95% and R2‐values>0.990) were used in the analysis. Expression changes in genes were determined via qPCR using a BioRad CFX96 instrument (BioRad) using cycling conditions of 95°C for 30 s, followed by 40 cycles of denaturing at 95°C for 10 s and annealing and extension at 60°C for 30 s, followed by dissociation curve analysis for all primer sets. qPCR analysis was performed in reactions containing SsoAdvancedTM Universal SYBR® Green Supermix (BioRad), 0.25 μM of each gene specific primer and 1 μl of cDNA. Data were analyzed with CFX Manager 3.1 software (BioRad) and ΔΔCt was calculated via normalization to the geometric mean of the relative quantities of the two reference genes 33. Data are depicted as fold change and percent reduction in fold change, as indicated, with *p*‐values calculated from paired *t*‐test of log_10_‐transformed relative expression values.

### Immunoassays

Mediator production in cell‐free CBMC supernatants was assessed via Bio‐Plex Pro Human 27‐plex immunoassay which measures IL‐1β, IL‐1RA, IL‐2, IL‐4, IL‐5, IL‐6, IL‐7, IL‐8, IL‐9, IL‐10, IL‐12p70, IL‐13, IL‐15, IL‐17, IFNγ, TNF, FGF‐2, G‐CSF, GM‐CSF, PDGF‐BB, VEGF‐A, CCL2, CCL3, CCL4, CCL5, CCL11, and CXCL10 (BioRad) and CXCL10, IL‐1Ra, VEGF (BioRad), IFNγ, and IFNα (eBioscience) Simplex assays. Samples were read using a Bio‐Plex 200 system (BioRad) and analyzed with Bio‐Plex Manager 6.0 software (BioRad). For statistical analysis, samples at or below the limit of detection were assigned the limit of detection value for the assay. Levels of pre‐formed, cell‐associated mediators were determined from freeze‐thaw lysates of CBMC. ELISA using commercial, matched antibody pairs was used to measure CCL8 (R&D Systems, Minneapolis, MN), CXCL9 and CXCL11 (both Peprotech).

### Statistical analysis

Data sets were analyzed using GraphPad Prism software version 6.0 (GraphPad Software Inc., La Jolla, CA, USA). Normally distributed data were assessed by paired *t* tests for 2‐group analyses or repeated measures ANOVA with Dunnett's post‐test compared to media control for >2 group comparisons. Data not normally distributed were analyzed by Wilcoxon signed rank tests for 2‐group analyses or Friedman's test with Dunn's post‐test, compared to media control, for >2 group comparisons. Values of *p* < 0.05 were considered significant.

## Results

### Human mast cells produce multiple mediators in response to type I and type II IFNs

**Figure 1:**
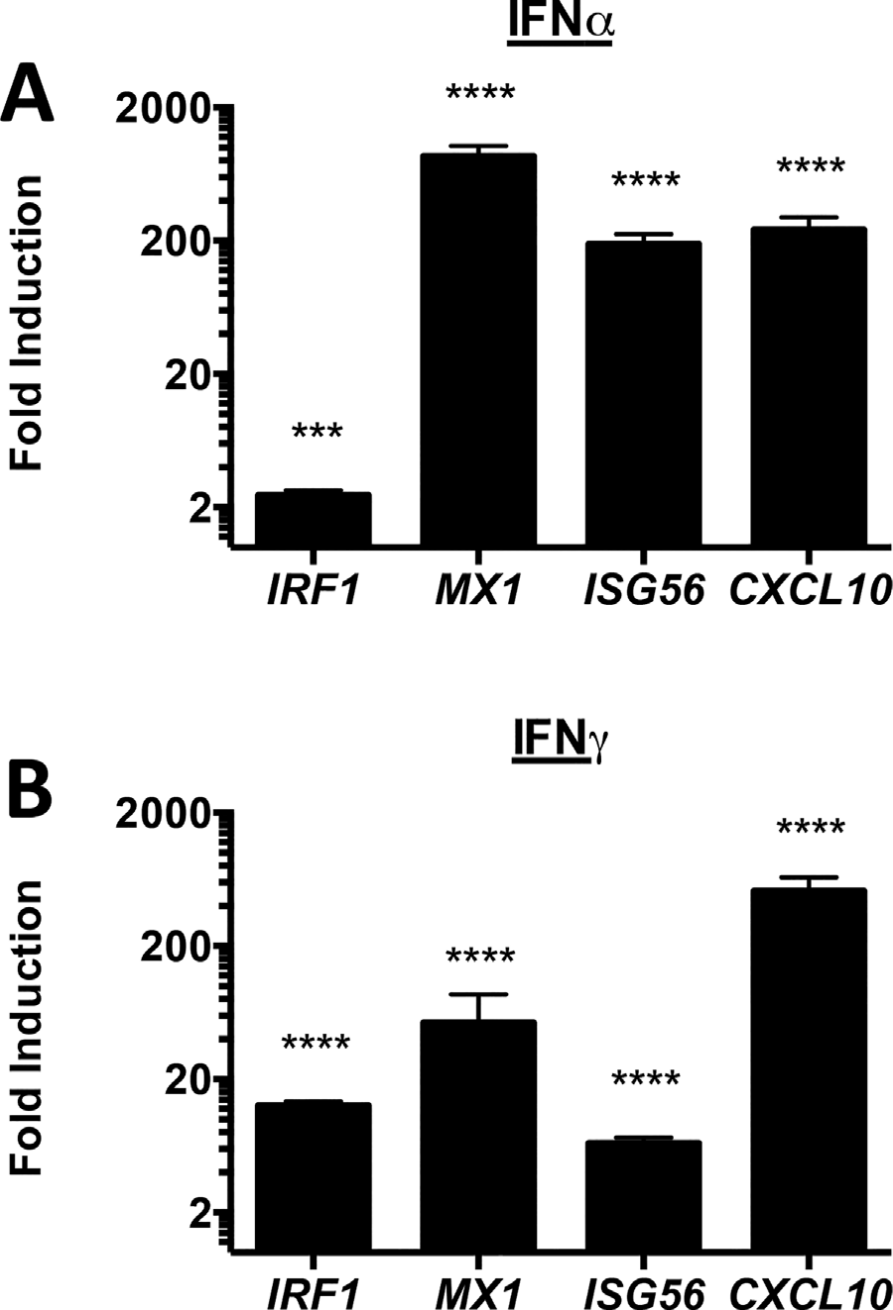
CBMC respond to type I and type II interferons. IFN response gene expression was assessed following 24 h treatment of CBMC (10^6^/ml) with A) 10 ng/ml (100 IU/ml) IFNγ (*n* = 10) or B) 5 ng/ml (100 manufacturer U/ml) IFNα2 (*n* = 5) for 24 h. Data are depicted as fold change over media control. *p < 0.05, **p < 0.01,****p < 0.0001, based on paired t‐test of relative normalized expression.

Functional responses of human mast cells following type I or type II IFN activation have previously been reported 34, 35. To characterize human mast cell responses to IFNs the expression of selected IFN‐inducible genes, in CBMC that were treated for 24 h with IFNα2 or IFNγ, were assessed (Fig. 1). The mRNA transcripts for the transcription factor *IRF1*, the dynamin GTPase *MX1*, the single strand RNA sensor *ISG56* and the IFN inducible chemokine *CXCL10* were significantly increased following treatment with either type I or type II IFN.

**Figure 2:**
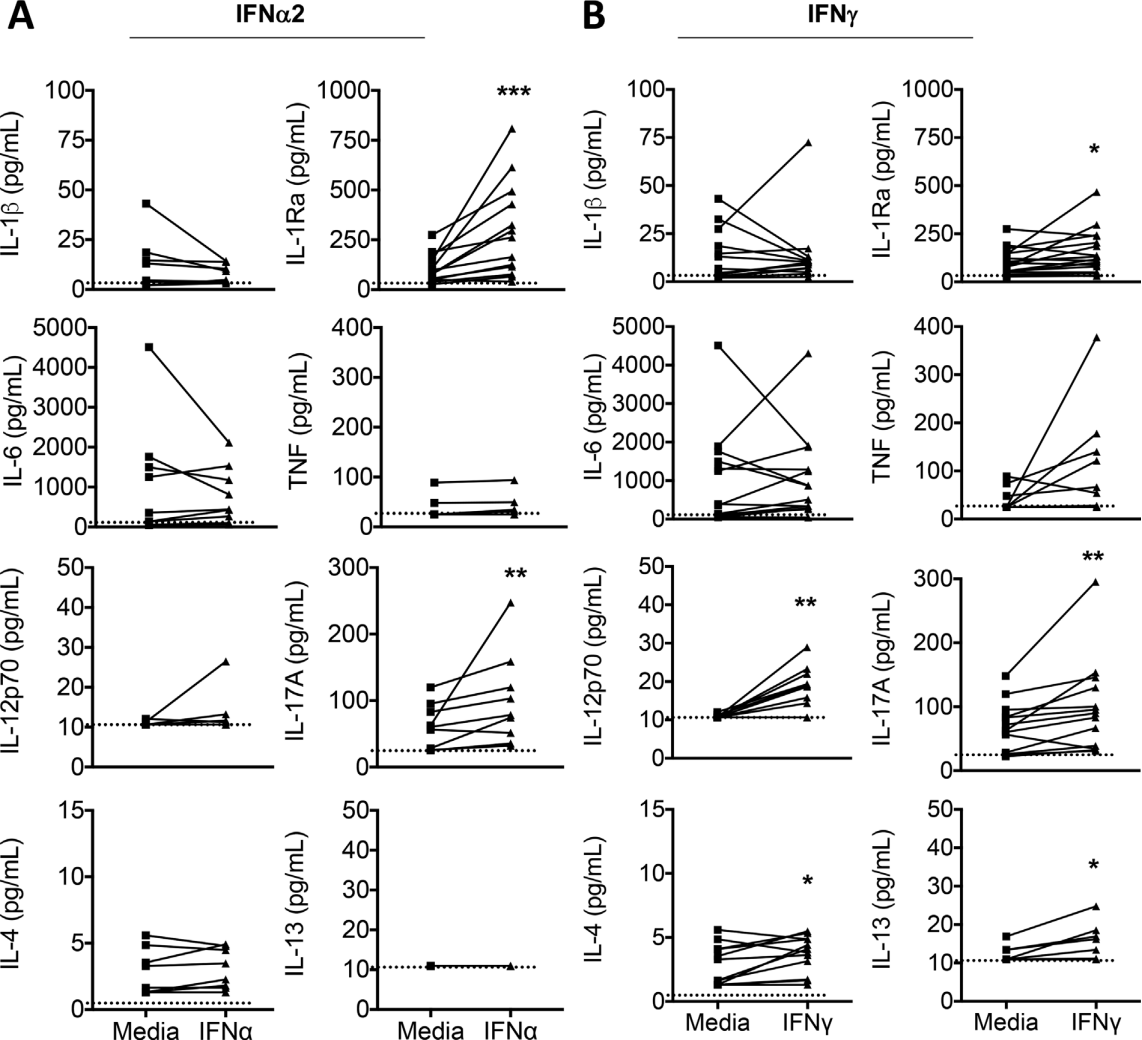
CBMC secrete a distinct cytokine response following activation with type I or type II IFN. The profile of cytokine production by CBMC (10^6^/ml) following 24 h activation with 100 IU/ml IFNγ (*n* = 17 for IL‐1Ra; *n* = 13 for all others) or 100 manufacturer U/ml IFNα2 (*n* = 13 for IL‐1Ra; *n* = 9 for all others) was determined using immunoassay. Each line depicts an individual CBMC sample. The limit of detection is depicted by hatched line. For statistical analysis, those samples with undetectable levels were assigned the limit of detection value. *p < 0.05, **p < 0.01, ***p < 0.001, ****p < 0.0001, Wilcoxon matched pairs signed‐rank test.

**Figure 3:**
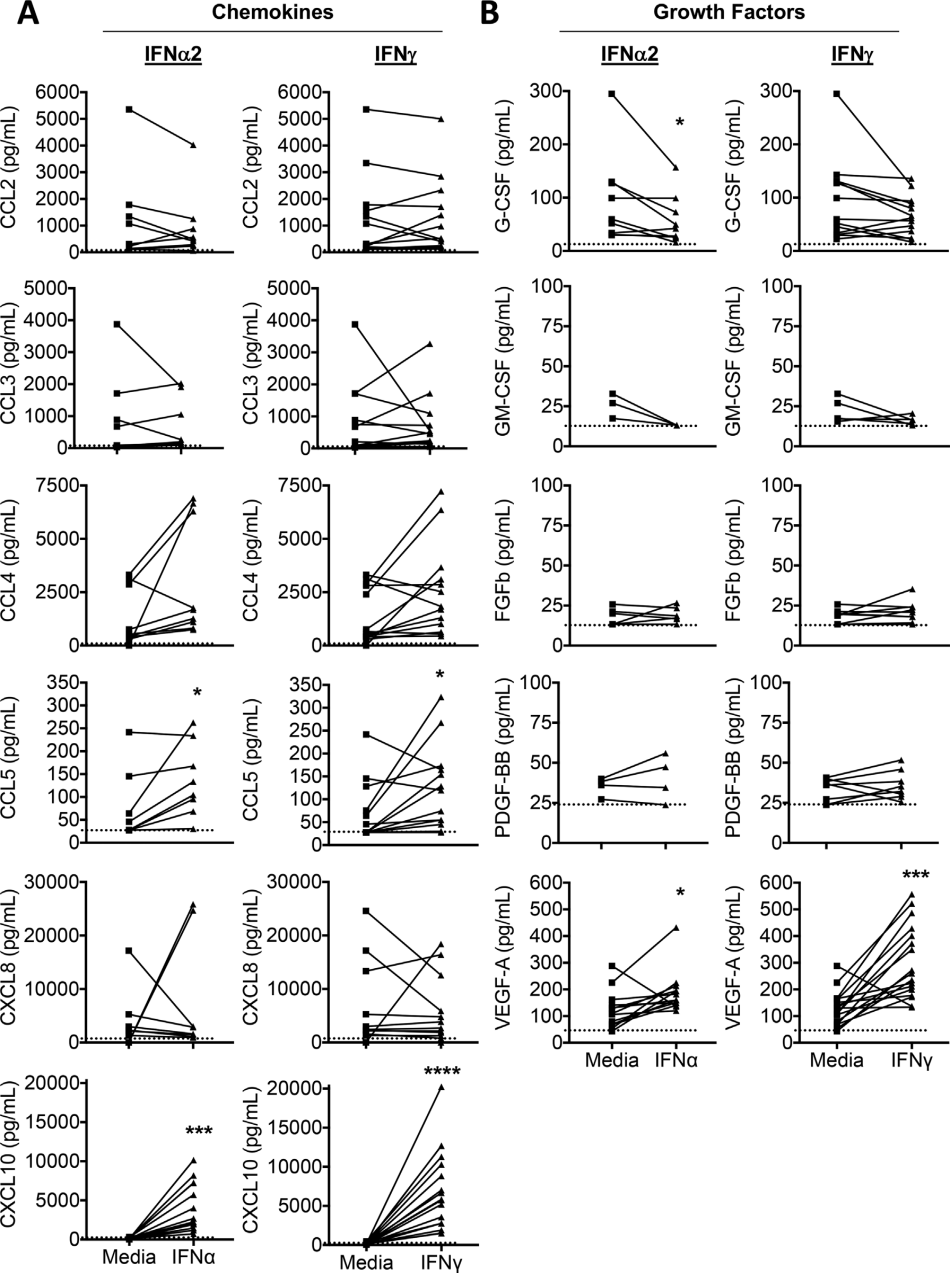
CBMC secrete a distinct chemokine and growth factor response following activation with type I or type II IFN. The profile of chemokine and growth factor production by CBMC (10^6^/ml) following 24 h activation with 100 U/ml IFNγ (*n* = 17 for CXCL10, and VEGF; *n* = 13 for all others) or IFNα2 (*n* = 13 for CXCL10 and VEGF; *n* = 9 for all others) was determined using immunoassay. Each line depicts an individual CBMC sample. The limit of detection is depicted by hatched line. For statistical analysis, those samples with undetectable levels were assigned the limit of detection value. *p < 0.05, **p < 0.01, ***p < 0.001, ****p < 0.0001, Wilcoxon matched pairs signed‐rank test.

The profiles of immune mediators released from IFNα2‐ and IFNγ activated mast cells were determined using a 27‐plex immunoassay (Figs. 2 and 3). Both IFNα2 and IFNγ induced the immunoregulatory factor IL‐1RA and IL‐17 (Fig. 2). IFNγ‐activated mast cells secreted elevated IL‐12p70 and such activation led to small but significant increases in released IL‐4 and IL‐13 (Fig. 2b). In contrast, IFN activation did not induce production of the classical pro‐inflammatory cytokines IL‐1β, IL‐6, or TNF from human mast cells (Fig. 2). Levels of IL‐2, IL‐5, IL‐7, IL‐9, IL‐10, IL‐15 were all below 20 pg/ml/million cells and were not induced following IFN treatment. As previously described 36, human mast cells secreted basal levels of CXCL8, however, this production was not increased following IFNα2 (*n* = 9) or IFNγ (*n* = 13) treatment.

Both IFNα2‐ and IFNγ‐activated human mast cells secreted abundant amounts of the T cell attracting chemokine CXCL10 as well as increased CCL5 (Fig. 3a). Secretion of the chemokines CCL2, CCL3, and CCL4 were not significantly increased in IFN‐activated mast cells (Fig. 3a). CCL11 was below the limit of detection for all samples (data not shown). CCL8 production was also assessed, via ELISA for IFNγ‐activated CBMC, and CBMC such cellssecreted abundant CCL8 (10,142 ± 112 pg/ml vs. 2811 ±136 pg/ml in diluent control, *p* = 0.031, *n* = 6).

Human mast cell production of selected growth factors was altered following IFN activation (Fig. 3b). Type I and type II IFN‐activated mast cells secreted significantly increased levels of the tissue remodeling factor VEGF. Mast cells spontaneously produced G‐CSF (92 ± 21 pg/ml, *n* = 13) and this production was decreased by approximately 40% following IFNα2 treatment (*p* = 0.039). There was also a trend toward decreased G‐CSF production in IFNγ‐treated mast cells (*p* = 0.068). In contrast, GM‐CSF, FGF‐2, and PDGF‐BB were produced at low levels (≤35 pg/ml) by unstimulated mast cells and their production was not altered by IFN‐activation (Fig. 3b).

### Mediator release from IFN‐activated human mast cells is degranulation independent

**Figure 4:**
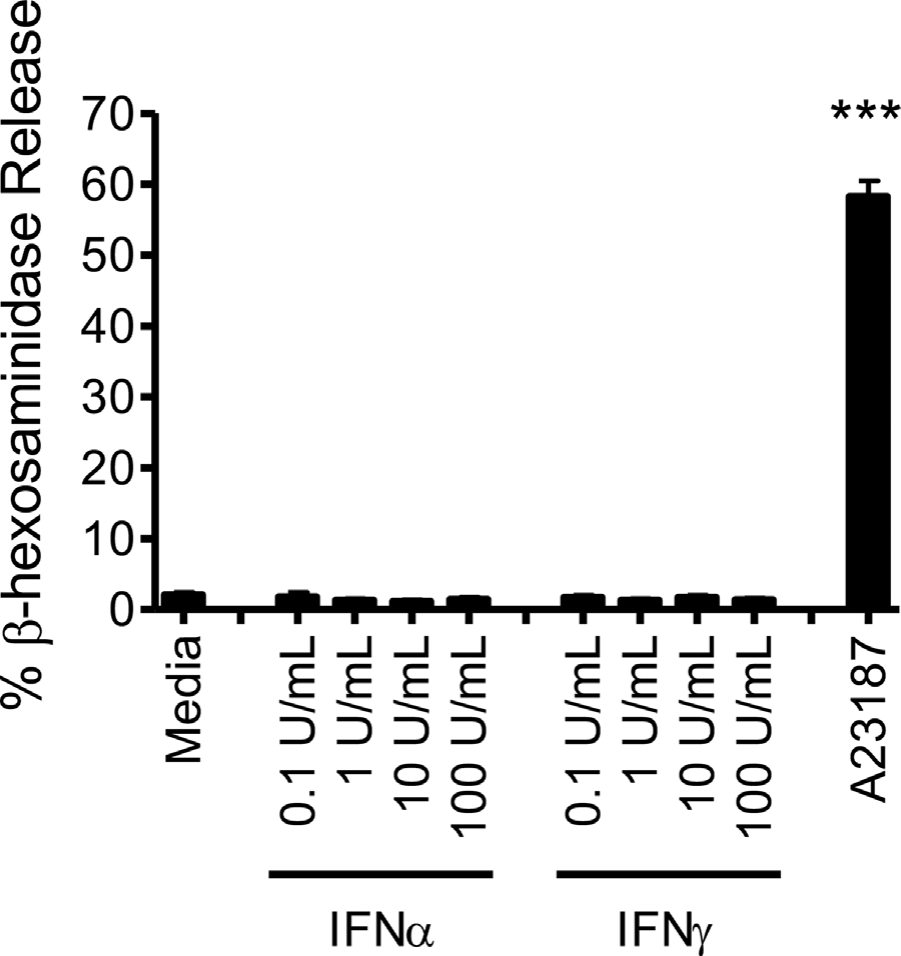
Type I and type II interferons do not induce short term CBMC degranulation. CBMC (0.5 × 10^6^/ml) in modified HEPES‐Tyrode's buffer were treated with increasing doses of IFNα2 or IFNγ or with the calcium ionophore A23187 (0.5 µM) for 20 min. The level of degranulation was assessed via β‐hexosaminidase release. Data depict the mean ± SEM for three independent experiments using three different CBMC cultures activated in triplicate. ***p < 0.001, paired *t*‐test.

To ascertain whether IFN‐induced mediator release from CBMC was attributable to activation‐ induced degranulation we initially performed multiplex immunoassay of human mast cell lysates to quantify the levels of pre‐formed cell associated cytokines. CXCL10 was undetected in CBMC lysates. In contrast, CBMC did contain small amounts of other mediators. IL‐1Ra, IL‐6, G‐CSF, and VEGF were all detected at amounts >15 pg/10^6^ cells (Table 1). To determine whether human mast cells degranulate in response to IFN activation we measured the amount of granule associated β‐hexosaminidase release following short‐term treatment of CBMC with increasing doses of IFNγ and IFNα2. All doses of IFNγ and IFNα2 examined induced <5% degranulation of CBMC (Fig. 4a).

**Table 1:** Levels of pre‐formed mediators in human CBMC^a^

Mediator	Concentration (pg/10^6^ cells)	(Range)	Mediator	Concentration (pg/10^6^ cells)	(Range)
*Cytokines*			*Chemokines*		
IL‐1β	8.5	(3.5–13.6)	CXCL10	ND	ND
IL‐1Ra	36.7	(18.5–50.5)	CCL2	8.7	(0.7–12.5)
IL‐2	ND^b^	ND	CCL3	0.4	(0.1–0.9)
IL‐4	0.2	(0.1–0.2)	CCL4	5.3	(2.1–10.1)
IL‐5	ND	ND	CCL5	2.9	(2.8–3.1)
IL‐6	17.9	(17.2–18.8)	CCL11	ND	ND
IL‐7	2.0	(1.2–3.6)			
IL‐8	7.0	(3.4–10.2)			
IL‐9	0.9	(0.9–1.0)	*Growth factors*		
IL‐10	ND	ND	FGF basic	ND	ND
IL‐12p70	2.6	(1.4–4.5)	PDGF‐bb	3.9	(2.4–8.0)
IL‐13	1.3	(1.1–1.9)	VEGF	59.0	(27.1–120.6)
IL‐15	ND	ND	G‐CSF	69.4	(48.8–90.2)
IL‐17	4.2	(2.6–5.6)	GM‐CSF	2.7	(2.4–2.9)
IFNγ	4.6	(4.1–5.3)			
TNF	ND	ND			

^a^ CBMC lysates (10 × 10^6^/ml) were prepared via freeze‐thaw and cell‐debris free supernatants were assayed for mediator production by multiplex immunoassay. Data depict mean concentration and range (*n* = 4). ^b^ND denotes content for all CBMC cultures was below the limit of detection of the assay.

### IFNs induce VEGF and IL‐1Ra from human mast cells in a dose dependent manner

**Figure 5:**
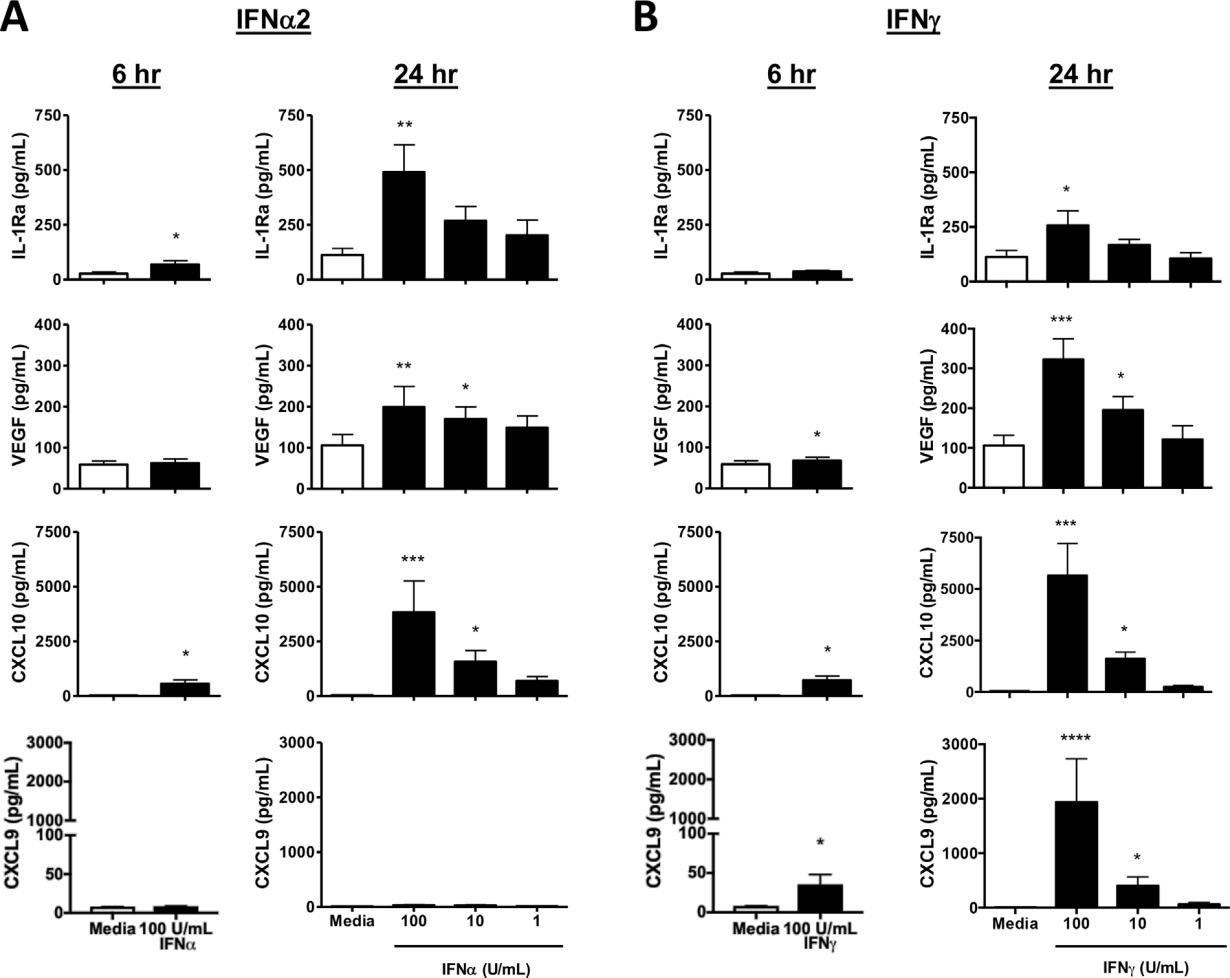
Dose and kinetic response of CXCL10, VEGF, IL‐1Ra and CXCL9 production by IFN‐activated CBMC. CBMC (10^6^/ml) were treated with increasing doses of A) IFNα2 or B) IFNγ. CXCL10, VEGF and IL‐1Ra production was assayed at 6 h and 24 h by multiplex immunoassay (*n* = 6) and CXCL9 production was assayed by ELISA (*n* = 8). *p < 0.05, **p < 0.01, ***p < 0.001, paired t‐test (6 hour) or Dunn's multiple comparisons post test compared to media control (24 h).

VEGF and the anti‐inflammatory factor IL‐1Ra have not been previously described to be produced from IFN‐activated mast cells and may be important for regulating local tissue remodeling and ongoing inflammation at sites of damage or infection. CXCL10 is a well‐defined part of the IFN response which often serves as a positive control for IFN stimulation. We further examined human mast cell responses to both IFNα2 and IFNγ at early (6 h) and late (24 h) time points and the dose dependence of VEGF, IL‐1Ra, and CXCL10 responses at 24 h (Fig. 5). IFNα2 and IFNγ induced all three mediators in a dose‐dependent manner. IFNγ, but not IFNα2, induced VEGF production at 6 h while IFNα2, but not IFNγ, significantly increased IL‐1Ra at the 6 h time point. Both type I and type II IFNs induced early production of CXCL10 by CBMC as early as 6 h but maximal responses were consistently observed at the later time point. The kinetics of production of additional CXCR3 ligand family members was assessed. CXCL9 was produced, in response to IFNγ, with similar kinetics to CXCL10 (Fig. 5B). However, IFNα2 activation did not induce a statistically significant increase in CLCL9 production. CXCL11 production was was undetectable at 6 and 24 h for all doses of IFNs (data not shown). These findings are consistent with a requirement for de novo synthesis and secretion for the majority of the mast cell VEGF, IL‐1Ra, CXCL10 and CXCL9 responses to the IFN stimulation.

### Virally activated human mast cells secrete IL‐1Ra in a type I IFN‐dependent manner

**Figure 6:**
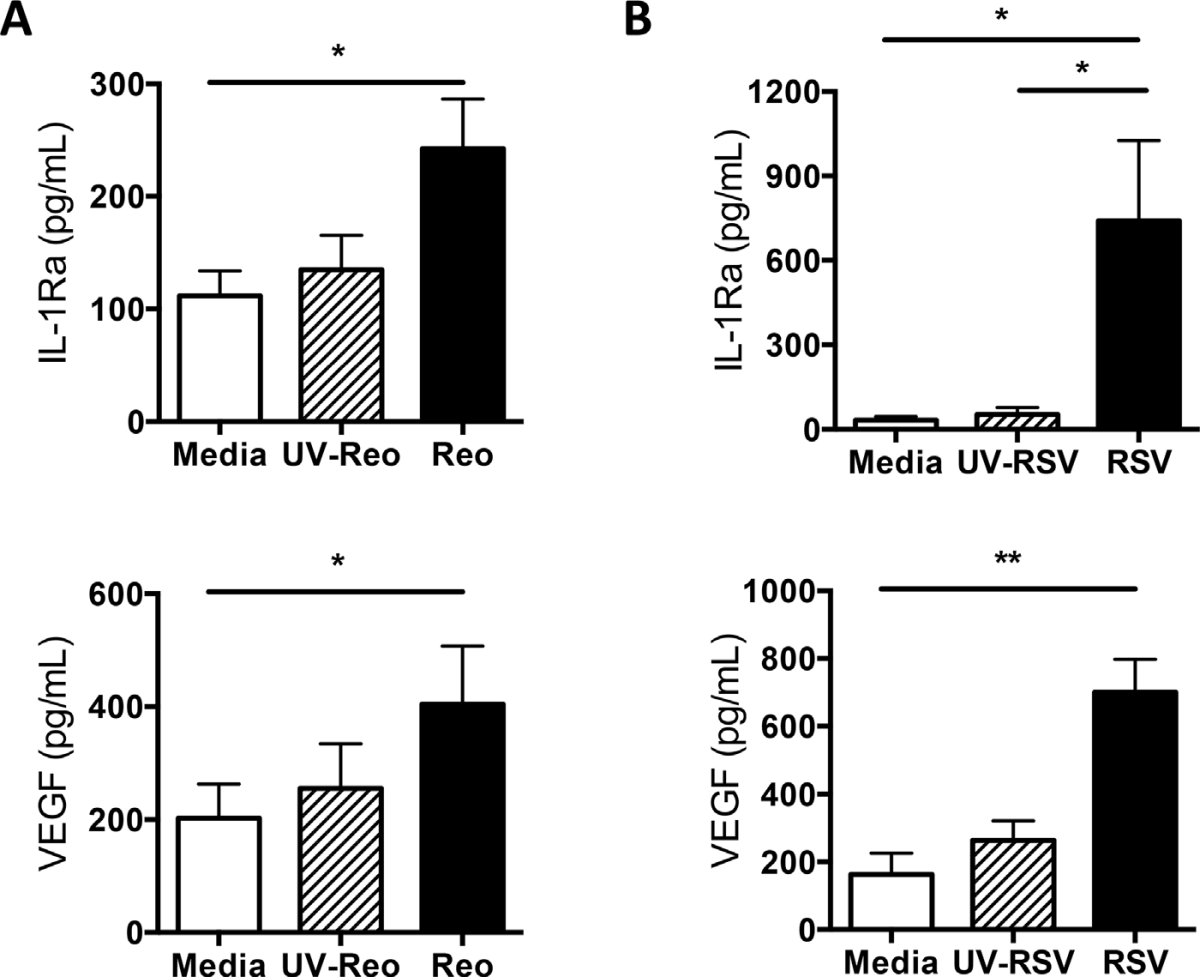
Virus‐activated human mast cells secrete IL‐1Ra and VEGF. CBMC (10^6^/ml) were incubated with (A) Live reovirus, UV‐inactivated reovirus and medium control or with (B) Live RSV, UV‐inactivated RSV and medium control, washed and further incubated for 24 h. IL‐1Ra and VEGF production was measured in cell‐free supernatants by multiplex immunoassay. *p < 0.05, **p < 0.01, Dunn's post‐hoc multiple comparisons test. *N* = 7 independent CBMC cultures for reovirus experiments, *N* = 6 independent CBMC cultures for RSV experiments.

IFNs are elevated in response to viral infection and are often critical for viral clearance. Reovirus is a ubiquitous virus that infects mast cell‐rich mucosal sites, and is rapidly cleared. It represents a useful model of effective anti‐viral immunity. Reovirus‐activated human mast cells secrete a wide range of type I IFNs 28, 37. To improve our understanding of the potential roles of mast cells as a source of IL‐1Ra and VEGF during viral infection, we evaluated mast cell production of these mediators following a viral challenge. Reovirus activated mast cells secreted increased amounts of both the immunomodulatory factor IL‐1Ra and the tissue remodeling factor VEGF‐A (Fig. 6A) when compared with controls.

**Figure 7:**
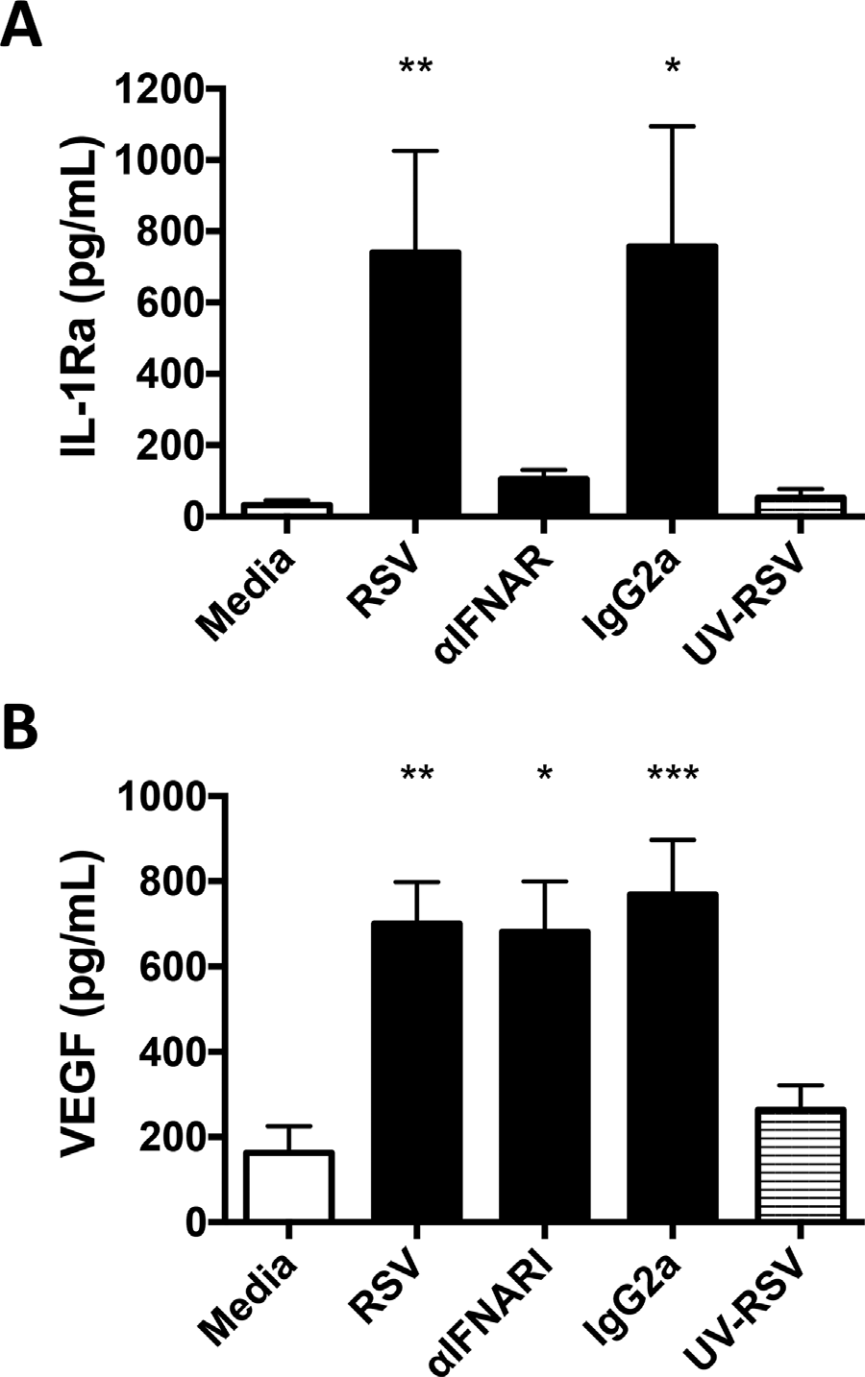
Effects of type I IFN blockade on RSV induced VEGF‐A and IL‐1Ra production by human CBMC. ^a^The profile of mediator production by CBMC (10^6^/ml) incubated with MOI 3–4 live (RSV) or UV‐inactivated (UV‐RSV) respiratory syncytial virus or with medium control for 24 h in the presence of media control or 5 μg/ml anti‐IFNAR or IgG2a isotype control was determined using immunoassay (*N* = 6). Differences between groups were assessed by Friedman's test with Dunn's post‐hoc test. *p < 0.05, **p < 0.01 compared to media control.

To assess the impacts of a more pathogenic RNA virus, mast cell responses to RSV were examined. RSV is a clinically important common RNA virus that is often associated with bronchiolitis and tissue remodeling events in the lung 38 and known to induce mediator production by mast cells 27. RSV‐infected human mast cells secreted high levels of both IL‐1Ra and VEGF (Fig. 6B). To assess the mechanism of RSV‐induced IL‐1Ra and VEGF we performed antibody‐blocking experiments to block the effects of type I IFN. RSV‐activated human mast cell production of IL‐1Ra, but not VEGF, was dependent on type I IFN (Fig. 7). Neither RSV, not reovirus induce significant short term degranulation of human mast cells, as we have previously reported. However both viruses induce substantial type 1 IFN responses at 24–48 h post infection 27, 28, 36.

**Figure 8:**
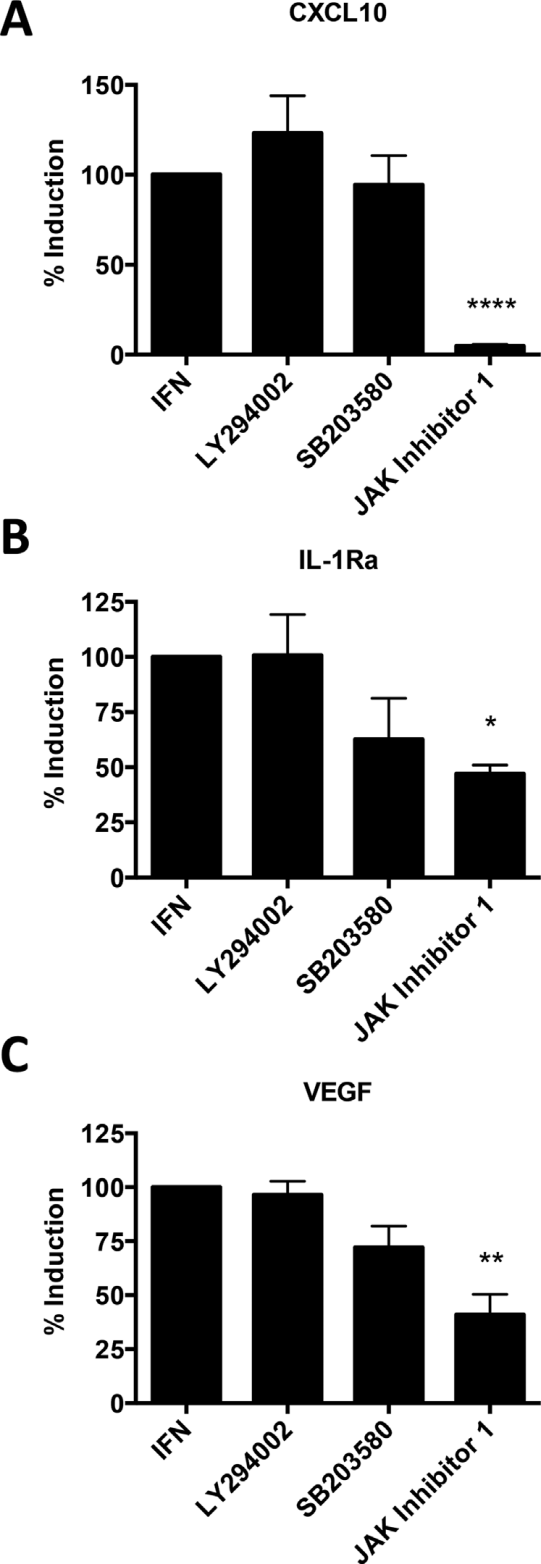
IFNα2 induction of CXCL10, IL‐1Ra and VEGF‐A is dependent on classical JAK/STAT signalling. CBMC (10^6^/ml) were pre‐treated for 1 h with 2 μM of the PI3 K inhibitor LY 294002, the p38‐MAPK inhibitor SB 203580 or with the JAK/STAT inhibitor JAK inhibitor 1, then incubated for 12 h with 5 ng/ml (equivalent to 100 manufacturer U/ml) IFNα2 or media control. CXCL10, IL‐1Ra and VEGF mRNA production was determined by qRT‐PCR. Data are depicted as percent decrease in fold change over media + vehicle control from IFN treated CBMC. ***p < 0.001, **p < 0.01, *p < 0.05, paired t‐test of relative normalized expression of IFN‐treated CBMC versus inhibitor‐treated CBMC (*n* = 3).

Human mast cells secrete type I IFN in response to several viruses, including Sendai virus 35, dengue virus 39, reovirus 37, influenza 37, and RSV 32, 37. Canonical IFN signalling is through the JAK‐STAT pathway. However, alternate signalling pathways, including MAPK and Pi3 kinase pathways, can additionally be regulated by IFNs and required for the induction of certain IFN‐induced genes 40. To examine the mechanism of IFNα2‐induced IL‐1Ra and VEGF production, we activated human mast cells with type I IFN in the presence of signalling pathway inhibitors. Mast cells were pre‐treated with 2 µM of either the PI3 K inhibitor LY 294002, the p38‐MAPK inhibitor SB 203580 or the JAK/STAT inhibitor JAK inhibitor 1. Pre‐treatment of CBMC with JAK inhibitor 1 significantly decreased mast cell production of CXCL10 (95% decrease), IL‐1Ra (53% decrease), and VEGF (59% decrease) (Fig. 8A). Expression of these mediators was not significantly altered by PI_3_ kinase (Fig. 8B) or p38‐MAP kinase inhibition (Fig. 8C).

## Discussion

Mast cells are rich at mucosal sites, such as the lung and gastrointestinal tract, which are prone to viral infection. They are found in large numbers at sites of angiogenesis and chronic inflammation such as the rheumatoid joint. IFNs are clinically used as immune modifying agents, but have a number of side effects including injection site reactions and impacts on the CNS. In order to harness the full potential of IFN‐based therapies and understand the role of IFNs in regulating mucosal immune responses, including allergy and asthma, it is crucial to understand their impact, beyond classical T cell mediated events. This study demonstrates that human mast cells exhibit a highly selective mediator response to IFNs marked by the selective production of certain chemokines, cytokines, and growth factors in the absence of degranulation. In keeping with the established role of mast cells as an early source of chemokines that can attract immune effector cells, human mast cells are a potent source of CXCL10 following type I and II IFN stimulation and also selectively produce VEGF and IL‐1Ra, suggesting important additional roles for IFN‐activated human mast cells in regulating immunity, inflammation, and tissue homeostasis.

CXCL10 is a chemokine secreted as part of a classical IFN response and was secreted by mast cells at high levels following activation with both type I and II IFN. A recent clinical trial suggests that inhaled IFNβ may be a potential treatment for virus‐induced deteriorations of asthma, particularly in difficult‐to‐treat asthmatics. Inhaled IFNβ enhanced local and systemic levels of CXCL10 22. Our study suggests that tissue resident mast cells might be important contributors to pulmonary CXCL10 production following type I IFN treatment. Production of the alternate CXCR3 ligand CXCL9 was also selectively induced by IFNγ treatment of mast cells. Mast cell production of CXCR3 ligands may be of particular importance in the airways. Airway smooth muscle associated mast cells have high expression of CXCR3 41. Thus, tissue resident mast cell production of CXCR3 ligands, in response to IFN, may increase mast cell numbers in reactive airways and have implications for asthma. Activation of human mast cells with IFNs induced significant increases in CCL5 (Figure 3) and CCL8. CCL5 and CCL8 are potent chemotactic factors for human NK and T cells with roles in asthma pathogenesis 42, 43, 44.

IFNs induced the release of selective cytokines and growth factors from human mast cells with known abilities to modulate immunity. IFN‐activated human mast cells secreted IL‐17A, which may have important roles in enhancing immunity. Indeed, IL‐17A and IFNγ can act synergistically to enhance macrophage function and inhibit bacterial growth 45. Minor increases in IL‐4, IL‐13, and IL‐12 were also observed following activation with IFNγ, but not IFNα2, demonstrating differential regulation by type I and II IFNs. Notably, in our in vitro system, some cytokines and chemokines, such as CXCL8, demonstrated high “background” levels of secretion even in the absence of IFN activation. Multiple older studies 1 have suggested that mast cells express a variety of cytokines and chemokines in vivo, but background levels of secretion have not been ascertained in human tissue.

Importantly, IFNs increased IL‐1Ra production by human mast cells without stimulating production of IL‐1β. Expression of the pro‐inflammatory cytokines TNF and IL‐6 were not significantly increased via IFN‐activation of human mast cells suggesting that IFN‐activated human mast cells may have an anti‐inflammatory role, which could limit the local activities of IL‐1. IFN‐activated human mast cells secreted VEGF, an important mediator of inflammation and promoter of angiogenesis. VEGF also has chemotactic activities for granulocytes and macrophages and can enhance matrix metalloprotease function 46. Thus, IFNs may enhance the tissue‐remodelling and host defence role of mast cells via increased production of VEGF. It is already recognized that human mast cells activated via PGE_2_ 47 or IgE/antigen 48 can produce VEGF. The ability of IFNs to induce a similar response from mast cells increases the possibility that mast cells have a critical local role in the interface between classical immune responses and the ongoing tissue remodeling that occurs following injury and infection beyond the acute setting of an allergic response. IFN modulation of VEGF expression has been examined in other cell type previously including peripheral blood mononuclear cells and melanoma call lines. However, IFNs were noted to decrease VEGF production in these settings. In additional preliminary studies we observed no increase in VEGF production by human epithelial cells (data not shown). These data suggest that the mast cell VEGF response to IFN activation may be unusual and tied to its key role in host defence and tissue remodeling.

Reovirus is a ubiquitous virus that infects mast cell rich enteric and respiratory tracts but is relatively benign in healthy children and adults 49. Thus, reovirus represents an excellent model to study effective, mucosal anti‐viral immunity. Reovirus infected mast cells produce substantial amounts of a wide range of type 1 IFNs, including IFNα2 28. Reovirus infected human mast cells recruit NK cells 36 and CD56^+^ T cells via distinct CXCL8 and CCR5 ligand dependent mechanisms 50. In addition to confirming that reovirus‐activated human mast cells are potent sources of several chemokines the current study demonstrates that reovirus‐activated human mast cells secrete elevated IL‐17A, TNF, IL‐1Ra, and VEGF. These mediators can regulate anti‐viral immunity, maintain tissue homeostasis and influence tissue remodeling.

RSV is a highly pathogenic virus associated with bronchiolitis, which is the leading cause of hospitalization of infants and young children worldwide 51. Increased susceptibility to RSV in early life has been attributed to decreased innate immunity. For example, RSV‐induced IFNα production by plasmacytoid dendritic cells is highly attenuated in term infants as compared to adults 52. Thus, IFNα appears to play an important role in anti‐viral immunity to RSV. Our previous results 32 and the current study suggest that mast cells are likely important contributors to this response. Human mast cells can secrete type I, II, and III IFNs 35, 39, 53 and may be a major source of type 1 IFNs during viral infection 28. We have recently identified that RSV‐activated human mast cells secrete type I IFN and release CXCL10 in a type I IFN‐dependent manner 32. This study demonstrates that RSV‐activated human mast cells also secrete IL‐1Ra in a type I IFN‐dependent manner. Thus, RSV‐induced IFN production by human mast cells can enhance their sentinel role and also function to inhibit IL‐1 mediated inflammatory responses following RSV infection. While IFN can induce VEGF, RSV‐induced VEGF was not dependent on type I IFN receptors indicating the presence of alternate VEGF production pathways during this infection. These could include secretion of preformed VEGF from human mast cells.

Mast cell responses to IFNγ may be particularly important in the context of mast cell‐NK and mast cell‐T cell interactions. NK cells and T cells are abundant sources of IFNγ in response to infection 12 which can be recruited in response to virus‐infected mast cell products or IFN. Mast cells are also a major source of type I IFNs which can promote NK cell activation and cytotoxic functions following viral infection. Epithelial cells, which are in the front line for viral attack, can also produce IFNs very early in the infection process. This might provide opportunities to limit viral infection at its earliest stages.

Mast cells are known sentinel cells that can be activated via multiple mechanisms and multiple cell surface receptors. These include hypoxic tissue microenvironments, complement receptors, toll‐like receptors and other innate immune recognition receptors 2. IgE mediated activation of dendritic cells has been associated with enhanced IFN production in systemic lupus but limits IFN expression following TLR‐mediated activation 54, 55, 56. These findings suggest a potentially complex relationship between IgE, dendritic cells, mast cells, and IFNs in disease.

Overall, we have demonstrated that human mast cells selectively secrete specific cytokines, chemokines, and growth factors in response to type I and II IFNs and IFN‐inducing viral infection. Thus, IFNs enhance the sentinel role of mast cells. The novel observation of enhanced VEGF and IL‐1Ra production suggests mast cell participation in modulation of pro‐inflammatory IL‐1 responses and tissue remodelling events. The impact of mast cell responses to IFN on the therapeutic function of IFN or on the development of side effects at mast cell rich sites, such as local inflammatory responses and sterile abscesses at IFN skin injection sites clinically remains to be determined.

## Acknowledgements

The authors wish to acknowledge the expert technical assistance of Nong Xu and Kristen Hunt and the assistance of members of the Department of Obstetrics and Gynecology and IWK Birth Unit for obtaining cord blood samples. This work was supported by the Canadian Institutes of Health Research (Award #'s MOP10966 and THC135230).

## Conflict of Interest

The authors have no conflicts of interest to declare.
